# Food Supply Impacts and Solutions Associated with the COVID-19 Pandemic: A Regional Australian Case Study

**DOI:** 10.3390/ijerph19074116

**Published:** 2022-03-30

**Authors:** Stephanie Louise Godrich, Flavio Macau, Katherine Kent, Johnny Lo, Amanda Devine

**Affiliations:** 1 Centre for People, Place and Planet, Institute for Nutrition Research, School of Medical and Health Sciences, Edith Cowan University, Bunbury, WA 6230, Australia; 2 School of Business and Law, Edith Cowan University, Joondalup, WA 6027, Australia; f.macau@ecu.edu.au; 3 School of Health Sciences, Western Sydney University, Campbelltown, NSW 2560, Australia; k.kent@westernsydney.edu.au; 4 School of Science, Edith Cowan University, Joondalup, WA 6027, Australia; j.lo@ecu.edu.au; 5 Institute for Nutrition Research, School of Medical and Health Sciences, Edith Cowan University, Joondalup, WA 6027, Australia; a.devine@ecu.edu.au

**Keywords:** food supply, COVID-19, local food, solutions

## Abstract

This study aimed to explore how food supply chains were impacted by COVID-19 and identify how the region could be better prepared for future crises. An online survey was completed by 107 consumers. In-depth interviews were conducted with 27 food supply stakeholders working in food production, distribution, retail, hospitality, institutions (i.e., childcare), logistics/freight and local government. Pre-COVID-19, farmer-direct distribution options and hospitality businesses comprised a substantial proportion of local food producer businesses. During the COVID-19 pandemic, consumers favoured local food supply options, farmers collaborated, and produce usually destined for export was redirected into local markets. Critical food supply actions included keeping borders open to food freight, enhancing social capital through real-time business communication, and business flexibility. Solutions included business adaptation, for example, farmers selling produce boxes and hospitality businesses selling excess stock, COVID-safe delivery, and collaboration through digital networks. To better prepare the region for future crises, actions to support communities could include a community approach to agriculture, increasing food supply diversity, facilitating transport to aid food distribution and purchasing, and more effective messaging to discourage panic buying. Actions to support retailers could include increasing access to wholesalers through online platforms. Actions to support producers could include improving infrastructure, such as more regional distribution facilities.

## 1. Introduction

The first confirmed case of the novel coronavirus (COVID-19) in Australia occurred on 25 January 2020 [1]. By March of that year, panic buying had peaked in Australia, with perishable and non-perishable goods purchasing increasing by 10.5% and 6.9%, respectively [2]. In consequence, many supermarkets subsequently placed purchasing limits on a range of food items [3]. These items were typically “staple” foods, including pasta and rice [3]. Despite these statistics, the Australian Government Department of Agriculture, Water, and the Environment asserted that any concerns about Australia’s food supply were “misplaced” with “access to a wide variety of healthy and nutritious food” [4]. However, it remains unknown how the COVID-19 pandemic impacted regional food supply chains and the effects of subsequent solutions that were created to mitigate such impacts.

Regional Western Australia (WA) has presented a unique context for food supply during the COVID-19 pandemic. With the challenge of the distance between food outlets, people living in regional locations ordinarily face greater challenges accessing healthy, affordable food than their urban counterparts [5,6]. Recent research has though indicated that many supermarkets adequately met consumer demand for food in pre-COVID-19 times with strong “local loyalty” [7]. However, the COVID-19 pandemic has exacerbated food supply issues, with research from the U.S. and Australia reporting problems such as lag time between food orders and supply. The “just-in-time fulfillment” of food stocks presented issues when supply chains were inflexible and unable to respond to unprecedented demand. Thus, supply chain vulnerabilities were highlighted and resulted in empty supermarket shelves [8,9]. Businesses sought alternative suppliers, food prices increased, many hospitality businesses closed, the media spread misinformation, and consumers sought alternative food suppliers to meet their needs [7,10]. Government-imposed restrictions on the movement of goods, services and people were additional challenges [11]. These issues may be particularly challenging for vulnerable people, defined as “individuals who may be unable to protect themselves against harm or exploitation” [12]. Conversely, a business-as-usual approach was adopted by some food supply stakeholders, with the advent of new products mitigating some supply issues. From the consumers’ perspective, many consumers purchased their food from local outlets or via online platforms as opposed to shopping in other regions [7,10]. Region-based or local food purchasing is a potential solution to food supply issues, particularly during crises such as the COVID-19 pandemic. Recent evidence suggested that consumer demand resulted in surges in local food sales at farm gates or farmers’ markets [9,13]. In regional WA, some consumers preferred to shop at smaller outlets and roadside stalls instead of the large supermarkets [9]. These purchasing behaviours were largely motivated by altruistic reasons; consumers reported wanting to support their local farmers. It has been suggested that regions and communities with strong social capital, encompassing trust, reciprocity, and social networks [14] have been more resistant to shocks to the food supply during the COVID-19 pandemic [15] and can be protective against food insecurity [14]. Yet, more must be known about how the COVID-19 pandemic impacted regional WA. Understanding how COVID-19 has impacted food supply in regional WA is important, given that regional businesses and community members are more vulnerable to food system impacts, to adequately prepare for inevitable future crises.

Therefore, the objectives of this study were to: (i) understand which food supply chains were utilised in South West Western Australia pre-COVID-19; (ii) understand how COVID-19 impacted the South West Western Australian food supply; (iii) identify critical factors associated with maintaining the South West Western Australian food supply during the COVID-19 pandemic; (iv) ascertain food business solutions implemented during the COVID-19 pandemic; and (v) identify how South West Western Australia could be better prepared for future crises.

## 2. Materials and Methods

In this paper, we will present a case study on food supply within South West WA, using a constructivist worldview. We have reported the methods and results sections according to the consolidated criteria for reporting qualitative research (COREQ) checklist [16].

### 2.1. Setting and Recruitment

**Figure 1:**
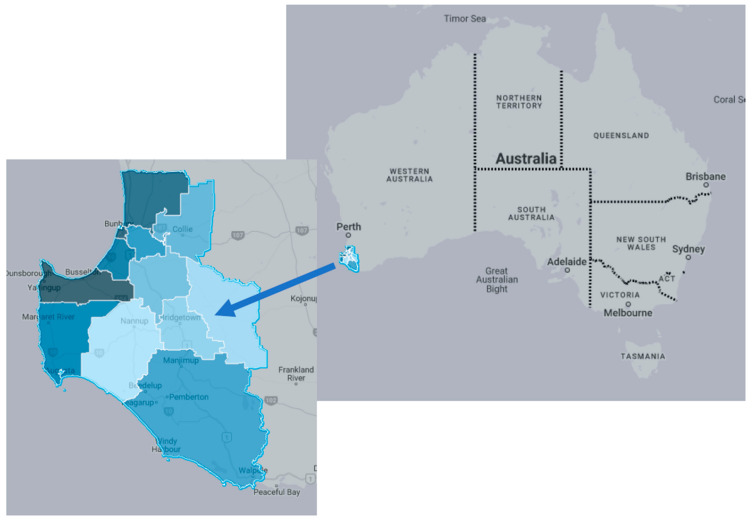
South West Western Australia (Adapted from REMPLAN MapBuilder, https://app.remplan.com.au/southwestregion/community/summary?state=vBxgT5VD1s37YrOhamMqmOI9tBtmDY, accessed on 1 February 2022).

The South West region of WA is approximately 24,000 square kilometres in area (Figure 1) [17], with the major town of Bunbury located approximately two hours south of Perth city [18]. A total of 181,801 people live in the South West region, where the median age is 41 years. Families in this region are mostly comprised of two people, with 23,053 families of this structure. Approximately one-quarter (26.1%) of residents were not currently in the labour force [18]. The South West region includes horticulture and agriculture industries, with respondents in previous research placing high importance on purchasing regionally grown fruit and vegetables. Key enablers of regionally grown produce included financially supporting local farmers (94% of respondents) and the local community (91% of respondents) [19]. Top barriers were seasonal availability (26% agreement), the perception that regionally grown produce was expensive (12% agreement), and food budgetary reasons (10% agreement) [19].

In our multi-phase study, resident South West WA (SWWA) adults aged 18 years and over were invited to participate in a cross-sectional consumer survey completed online (Phase 1). We engaged a professional list broker who was provided with inclusion criteria of: adults aged over 18 years, who had email addresses in the “South Western/South West area” in postcodes 6172 to 6176 and 6207–6398 and had opted into being contacted for research and marketing purposes. Email addresses on the provided database were emailed directly by the chief investigator, including an invitation letter. Additional recruitment strategies included emails to community resource centres and promotion via a university staff directory, website, and newsletters. Social media platforms including Twitter, LinkedIn, and Facebook were used as additional recruitment methods, with posts promoted via paid boosting. No incentives were offered for the study.

Food supply stakeholders were purposively sampled for the interview study phase (Phase 2), using a contacts database generated by the investigator that included *n* = 145 contact details for SWWA aged care, hospitality, childcare, independent schools, farm businesses, farmers’ markets, retail outlets, transport/freight companies, and relevant state and local government agencies. These contact details were sourced from an Internet (Google) search. The database listed details including the business and contact person’s name, telephone, and email address. All listed leads were invited to participate in the study via an emailed information letter and consent form, with a minimum of three contact attempts.

### 2.2. Survey Tool

The Phase 1 consumer survey included 29 closed- and open-ended questions. Questions included those on consumers’ opinions on how COVID-19 had impacted the Australian food supply, USDA Food Security Survey Module (6-item short form) [20] questions, whether the consumer had tried to purchase food items that were unavailable, and how South West food supply could be better prepared for future crises, among other questions. An exemplar question was: ‘Have you observed any innovative ways of maintaining the Australian food supply during COVID-19? (for example, businesses changing the way they sell food). If so, what are they?’. Thirteen demographics questions were included, such as age, educational attainment, and the number of adults and children residing in the household. A ten-question interview guide (Phase 2) was developed including the interviewee’s work role and time in the role, a pre- and post-COVID description of South West food supply chains, observations regarding consumer purchasing during COVID-19, important steps to maintain the food supply during the pandemic, and perceived food supply solutions. A series of prompts were included and used as needed.

### 2.3. Data Collection

Phase 1 consumer survey data were collected from 3 May to 14 July 2020 using Qualtrics (Qualtrics, Provo, UT, USA). Respondents were required to provide active consent before entering the survey by reading the information letter and consent form located on the front page of the survey. Respondents were unable to progress to complete the survey unless they clicked ‘I consent’. To collect Phase 2 interview data, two female project team members trained in qualitative data collection, one with a PhD, conducted stakeholder interviews between 11 May to 13 August 2020. Participants submitted a signed consent form prior to the interview, which was conducted via telephone. The interviewer did not have an established relationship with participants prior to the interviews. Interviews were between 20 and 80 min in duration (48 min on average). Each interview commenced with an overview of the research project and its aim. A ‘research journal’ entry was completed after each interview to capture key points discussed and assist with initial coding ideas.

### 2.4. Data Analysis

Survey data were exported from Qualtrics into SPSS version 26 (IBM Corp. Armonk, NY, USA), where data were cleaned and analysed. Eight responses were removed for analysis due to excessive (95–100%) missing data. Open-ended responses were additionally imported into QSR NVivo 12 (QSR International Pty Ltd, NVivo, Burlington, MA, USA, Release 1.0, 2020) where content analysis was undertaken by two project members. Open-ended question responses to the consumer survey were thoroughly read by one interviewer and compared with interview transcripts. Food supply stakeholder interviews were transcribed verbatim into Microsoft Word, de-identified, checked for transcription accuracy and imported into a separate QSR NVivo 12 Pro (QSR International Pty Ltd. Release 1.0, 2020) to the consumer survey responses. A thematic analysis was conducted by three project members to determine interview themes, as guided by Braun and Clarke [21]. Due to the similarity in content, the same nodes were created in both databases to enable comparisons between consumer and food supply stakeholder responses. Data were initially coded against the interview questions by one team member and, for each question, inductive nodes were created as themes discussed in relation to that question were identified. Nodes were then combined where they contained similar statements. An ‘analysis journal’ was kept within NVivo to document the development of nodes and themes. A coding framework included a summary of nodes and exemplar quotes. Codes were thoroughly checked by three team members. The first and second team members discussed initial code ideas, reviewed the coding frame and coding accordingly. A third team member reviewed NVivo codes to ensure agreement with the coding approach. Minor recoding amendments were subsequently made, such as creating new child nodes using an iterative process, combining and then deleting superfluous child nodes, and recoding some content to other nodes (see Table 1). A ‘rich, thick’ description of the whole dataset was undertaken to ensure transferability. A summary of results was presented in a public forum on 11 November 2020, with the opportunity for participants to refine project recommendations (not reported on in this publication). Ethics approval for this research was received from the Edith Cowan University Human Research Ethics Committee (approval number 2019-00966-GODRICH).

## 3. Results

Participant demographics are presented in Table 2. A total of 161 participants self-selected into the study by completing the online consumer survey with 107 complete responses captured. A total of 29 food supply stakeholders consented to participate in interviews, including primary producers (*n* = 7), local government community development or environmental health staff (*n* = 7), retailers (*n* = 4), childcare coordinators or cooks (*n* = 3), aged care hospitality coordinators (*n* = 2), logistics or freight managers (*n* = 2), open-air/farmers’ market managers (*n* = 2), and community food workers (*n* = 2). Those invited stakeholders that did not respond to the invitation either did not provide a reason, or cited time constraints as reasons for not participating. A total of 29 interviews took place. A total of 27 interviews were utilised in analyses due to audio device failure during two interviews. Box 1 provides an indication of a typical consumer respondent and typical food supply respondent in our study.

Box 1 **Typical consumer and stakeholder study respondents.**

Typical consumer respondent: female, lived at home with their partner, with no children residing in the household, had completed a TAFE or university qualification, were married, retired or unemployed, had a disability or health condition, and were the main household shopper.

Typical food supply stakeholder respondent: Worked in the retail sector, retail manager, with 6–10 years of experience in the field.

Consumers and food supply stakeholders provided insights into the South West food supply prior to and during the COVID-19 pandemic, in addition to business solutions borne from the pandemic. These respective insights have been compared below.

### 3.1. Stakeholder Perceptions of the Pre-COVID-19 South West Food Supply

Food supply stakeholders were asked to reflect on what the South West region’s food supply system looked like before the COVID-19 pandemic. They described farmer-to-consumer direct options, operating alongside longer food supply chains, the risks and accreditation requirements of food businesses, and the importance of collaborating with other food supply stakeholders. These insights have been described below:

#### 3.1.1. Local Customers (85 Coded Statements)

Before the pandemic, local restaurants comprised a large proportion of some producers’ businesses. Other farmer-direct distribution options included street trade stalls in town centres, multiple farmers’ markets, and direct deliveries by farmers to customers throughout local towns, or farm shops, e.g.,

*“We never really had an issue with the food supplies from the local farmer’s markets which were just seasonal foods like fruits and vegetables.”*

(Institution sector, aged care facility/hospitality coordinator)

#### 3.1.2. Corporate Customers (75 Coded Statements)

While some farmers sold directly to restaurants, others first sold to wholesale customers who on-sold produce to the hospitality sector. Local produce was sent to regional packing sheds, and then subsequently onto large wholesale markets in the capital city of Perth, throughout Western Australia, across Australia or internationally thereafter, e.g.,

*“It starts from the grower supplying the fruit to us … we pack the fruit and then we supply supermarkets. Most of what we supply goes into the eastern states…Probably 10 percent of what we do is export markets mainly into Singapore, Malaysia and Hong Kong.”*

(Freight/logistics sector, logistics and freight manager)

#### 3.1.3. Reputation, Accreditation (34 Coded Statements)

Growers were required to abide by a strict food safety and quality assurance program to reduce the risk of accidents or contaminated food. Aside from the time imposed, registration of such programs presented a significant expense for producers, with one example costing more than AUD $100,000 per annum. However, having such systems in place ensured contracts with larger retail outlets, e.g.,

*“…Lots of recording down to like how you measure scales in packing shed every hour to make sure they’re calibrated properly…so as a small [business]… it’s a bit hard…then have a third-party auditor come in. The audit itself is about eighteen hundred to two thousand dollars a year. And the testing and the paperwork you can imagine add up.”*

(Production sector, Producer)

Industry reputation was important; a recent bacterial outbreak within the rockmelon industry, and deliberate sabotage within the strawberry industry brought the reputation of their whole respective industries into question. Aged care homes and childcare centres were also subject to auditing and accreditation processes relating to food, which required substantial staff time to meet adherence requirements. Bureaucratic processes precluded such institutions sourcing local food, such as domestic chicken eggs, from local families wanting to contribute during a time of food shortage challenges.

#### 3.1.4. Cooperation, Collaboration (5 Coded Statements)

Producers worked in collaboration with chefs to incorporate produce into restaurant meals. Farmers’ markets ensured locally grown food was sold within the region, such as through stalls and value-added food businesses present, e.g.,

*“Would be predominantly showcased at those events by chefs, or leading people in the industries.”*

(Production sector, producer and retailer)

### 3.2. The Impact of COVID-19 on South West WA Food Supply

Following on from the picture of pre-COVID South West food supply chains, the impact of the pandemic on food supply has been outlined below. Both consumer survey open-ended responses and food supply stakeholder interview data have been compared to showcase different perspectives on the issues.

#### 3.2.1. Changes in Product Availability (52 Coded Statements)

Products such as fresh produce, meat, pasta and rice, and frozen vegetables were in limited supply. Stocks were “*just enough food for the nation with normal patterns and normal exports*” but were impacted when the resultant panic buying peaked. The purchasing limits applied to certain food products resulted in some community members “*going without*” food items ordinarily purchased.

Staple products, such as meat, “*spiked*” in price, e.g.,

*“We were looking for mince for some of the meals and if the supply hadn’t diminished, then the price had gone up so we could not get it at all or get it at almost a 200% price increase.”*

(Institution sector, institution coordinator or cook)

A reduction in food exports resulted in first grade produce suddenly being available within local supermarkets, while buyers from eastern Australia reportedly offered to purchase whole crops which was a rare experience for some farmers. Certain products including mince, flour, bread, and spinach were difficult to obtain. Consumer panic buying contributed to unreliable food availability, e.g.,

*“The panic buying led to a cycle where you would get a huge number of sales…and then everybody had their fridges full so would buy nothing for 10 days… I know the big packing sheds, a couple of times had huge orders put to them by the big supermarkets only for them to be cancelled.”*

(Production sector, primary producer)

#### 3.2.2. Changes in Distribution Modes (46 Coded Statements)

An increased reliance on locally sourced food occurred due to challenges acquiring imported food through disrupted supply chains. Local producers diverted sales, typically destined for export, into their local market. Some businesses experienced customers vanishing “*overnight*” due to restaurant closures. According to one interviewee, the hospitality sector downturn reportedly cost several producers between 30–80% of their business income. Considering hospitality business closures, direct supply chains were optimised, including home-delivered produce boxes or meal kits advertised through social media and sold. Some farmers’ markets were required to close, despite being deemed an essential service, as a result of concerned patrons or patrons disobeying social distancing procedures. Online farmers’ markets countered this problem for some organisations. Within institutions, childcare workers described the daily challenges they faced when feeding children, e.g.,

*“I basically was having to take it day by day, as to how I was actually going to be preparing any meals … just walking around the shopping centre trying to figure out what I was going to cook on that particular day.”*

(Institution sector, institution coordinator or cook)

#### 3.2.3. Changes in Habits and Processes (32 Coded Statements)

Imported produce sales declined in favour of more locally or home-grown food. Some community members who experimented with home food growing did so until supermarket supplies had improved. Smaller independent food outlets and supermarkets gained popularity among consumers; some independent retailers increased sales by 30%, according to one interviewee. COVID-safe practices, such as removing food tasting options, were implemented at farmers’ markets. Many farms also enhanced their safety practices, including increasing hand sanitiser provision and moving to online payment forms, to ensure consumer safety. Some farmers ceased farm gate sales, as a strategy to protect their employees from potentially contracting the virus:

*“Farm gate sales … ceased completely because when the outbreak happened, say, for instance, we’re only relatively small property, but we still had $160,000 worth of gross apples to pick… gross value of apples to pick for the season…We made the decision at that point not to allow anybody but the workers onto the property…It’s not worth the risk of getting the workers sick or ourselves sick.”*

(Production sector, primary producer)

### 3.3. Critical Steps to Maintain the South West Food Supply during the COVID-19 Pandemic

Food supply interviewees’ reflections of key steps to maintain supply through the SW region are described below.

#### 3.3.1. Keeping Supply Chain Infrastructure Operating (37 Coded Statements)

Allowing food supply workers to pass through intrastate border closures was an essential step to maintain food and fuel supplies within the SW region. For example, fuel, warehousing, refrigeration and food distribution throughout the region were largely unaffected from a supply infrastructure perspective. The continuation of food freight provided customers with access to fresh produce through the food supply:

*“If they closed that [borders] down, that would have been a huge problem. But they didn’t because they couldn’t stop the food coming through, people still have to eat.”*

(Freight/logistics sector, logistics or freight manager)

Further, keeping wholesale markets open, categorising farmers’ markets as essential services and allowing home deliveries of food, particularly for vulnerable (e.g., younger adults, older adults, refugees, Aboriginal people, females and university students), ill, or immobile people, “*kept a lot of businesses afloat*”. The Australian Government’s JobKeeper scheme (federal government payment support scheme for Australian businesses) likewise assisted businesses to keep operating. The opportunity to retain on-farm workers was paramount to continuing business operations, albeit it was a case of luck that *“there were a lot of backpackers and workers already in town”*.

#### 3.3.2. Effective Communication (13 Coded Statements)

Communication was challenging, during the rapidly evolving pandemic situation, with direct messaging and telephone communication methods to ensure producers “*were at the forefront of what they [consumers, wholesalers] wanted*”. Large food supply businesses and buying agents offered regular updates regarding food stocks online via a portal to ensure information dissemination was timely and accurate for food retailers. Some local governments published weekly updates on their website to inform the community about where they could access emergency food relief. In addition, they acted as critical support mechanisms to guide business owners through COVID-safe plans:

*“A lot of them wanted to speak to a person, they spoke to a Shire and one of their health officers …and we’d sort of [be] talking through the COVID plan and how to fill it out… I guess we did all the legwork for what the state government was, was actually implementing in terms of the directions. So, make them feel comfortable and that they could be confident what they’re doing is the right thing.”*

(Government sector, local government community development or environmental health staff)

#### 3.3.3. Business Adaptability (13 Coded Statements)

Multiple contingency plans were drafted and often combined during the first wave of the pandemic, given food supply uncertainty. Some hospitality businesses sold excess stock, such as flour, to vulnerable groups to reduce waste, while some retailers drove up to 90 min to deliver local produce to keep their businesses operating. Businesses pivoted, implementing ‘safe to fail’ strategies to ensure adequate supplies. Institution cooks adapted recipes, “*thinking on their feet*” with available food items, while producers adopted new ways of selling their produce, e.g.,

*“If I use example of getting into food boxes, it was an experiment and we had no idea what was going to happen. It’s been a huge success in working with other farmers…we weren’t thinking about it eight weeks ago.”*

(Production sector, primary producer)

Emergency relief adapted their food provision, with support from local and federal governments, utilising simple food vouchers that could be obtained in large quantities.

### 3.4. Food Business Solutions Borne from the Pandemic from Consumer and Stakeholder Perspectives

Consumers and food supply stakeholders reported on solutions they had witnessed during the pandemic, relating to food businesses and fresh produce food supply. Their respective responses have been tabulated below (Table 3) and highlight the value of business adaptability, producer-to-consumer direct sales, and collaboration to maintain business operations. For example, cafes adapted their business model to sell staple foods, including eggs, bread and milk, and some school canteens sold family meals. Contactless sales methods were created, such as online ordering and drive-by collection, and increased in popularity. Food producers collaborated to increase buying power to offer consumers lower priced produce.

### 3.5. How South West WA Could Be Better Prepared for Future Crises

**Figure 2:**
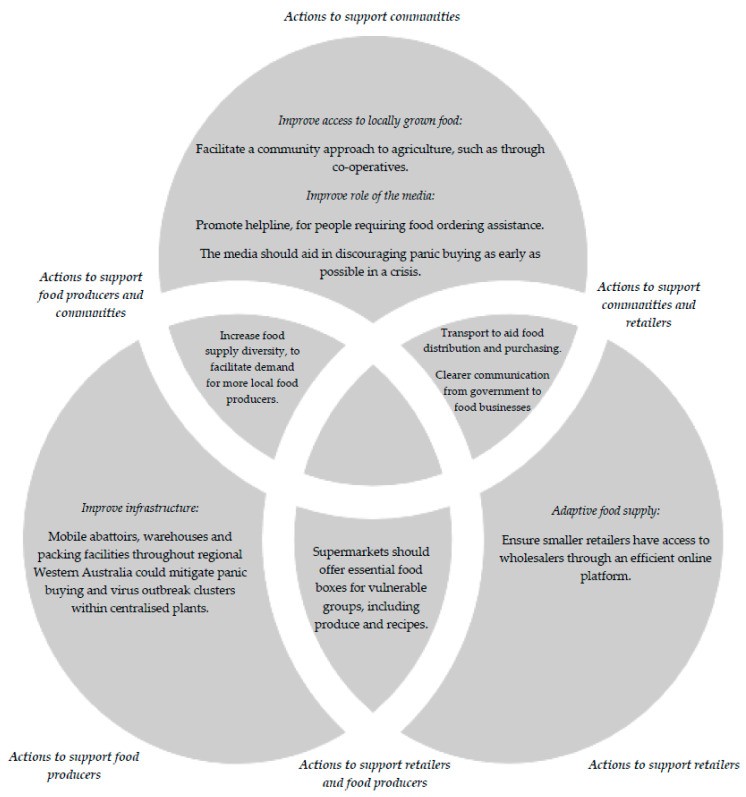
Food producer, community and retailer recommended actions to prepare the South West region for future crises.

Both consumer and food supply stakeholder respondents provided their opinions regarding how the region could prepare for future pandemics and crises. The collective responses relating to infrastructure, skill-building, food purchasing and food supply adaptations, and improving the role of the media have been synthesised in Figure 2. In summary, actions to support communities could include facilitating a community approach to agriculture (i.e., through co-operatives), and improving the media’s role in pandemics, such as through more effective public health messaging, to discourage panic buying. Actions to support food producers could include improving infrastructure, such as creating mobile abattoirs or food distribution centres in regional locations:

*“Mobile abattoirs to allow access to local meat protein if bulk transport and processing is impacted.”*

(Consumer respondent)

Actions to support retailers could include ensuring smaller retailers have access to wholesalers through an online platform. Cross-over actions to support food producers and community members could include increasing food supply diversity. Cross-over actions to support both communities and retailers include transport to aid food distribution and purchasing and clearer government communication to food retailers, regarding lockdowns or operating restrictions, e.g.,

*“Government actually giving notice of changes to food retailers or upcoming changes, having a plan for this kind of situation. For example, I bought $5 K of food stock for three cafes in the preceding two days, much had to be frozen (lower quality) or preserved or thrown away. Very badly handled when they must have had a pandemic plan to refer to.”*

(Consumer respondent)

## 4. Discussion

The objectives of this study were to: (i) understand which food supply chains were utilised pre-COVID-19; (ii) understand how COVID-19 impacted SWWA food supply; (iii) identify critical factors associated with maintaining the SWWA food supply during the COVID-19 pandemic; (iv) ascertain food business solutions implemented during the COVID-19 pandemic; and (v) identify how SWWA could be better prepared for future crises.

Our interview participants described a pre-COVID-19 food supply landscape that included local restaurants and farmer-direct options, such as farmers’ markets. Wholesale customers distributed fresh produce across the state and Australia. Food safety risks were mitigated by strict on-farm and within-institution quality assurance programs. Our findings were supported by the Australian Government’s FOODmap report, which outlined an increasing interest among consumers for local food supply chains, and alternative food distribution channels, such as eating-out options [22]. Similarly, in the U.S., food-away-from-home represented around half of consumer food purchases [23], with farmer-direct purchasing methods, such as farmers’ markets, farm gate sales and pick-your-own, popular with consumers [6].

In our study, the COVID-19 pandemic increased reliance on local producers and smaller food outlets given the downturn in food exports. This was also reported globally [24,25]. Producers were required to pivot their businesses to attract other customers with the closure of hospitality businesses. Our findings relating to the downturn in hospitality businesses have also been reported in Germany and Slovenia, with 22% of participants reporting they ate at cafes prior to the lockdowns [26]. In France, almost half (40.4%) of one study’s respondents reported they no longer ate out of the home [27]. In the U.S., food-away-from-home sales substantially decreased in favour of supermarkets and other retailers [23]. Globally, agricultural commodity prices have decreased by 20% during the pandemic as a result of the downturn in restaurant demand [28]. However, some businesses have adapted to mitigate a loss in profits. A large UK fast-food chain adapted its business model to convert 65 restaurants into shops selling ready-made refrigerated meals [29]. In our study, we found that during lockdown periods, home-delivered produce boxes, meal kits, and online orders surged in popularity. These findings are echoed internationally with online food companies experiencing unprecedented demand [6]. For example, in the U.S., online ordering surged from 39% pre-pandemic to 79% of consumers reporting this method of food purchasing during the initial onset of the pandemic [12]. However, this has resulted in delivery issues [29]. Costa Rican research found that direct sales, including text messaging, direct messaging via social media, and web platform sales, dominated sales methods for farmers as a result of COVID-19 [30]. However, farmers less familiar with technology-based sales reported challenges, such as a lack of time, increased costs, and a lack of skills required to make such sales [30].

Critical factors associated with maintaining the SWWA food supply during the COVID-19 pandemic were reportedly containing the virus, achieved through lockdowns and social distancing rules. Importantly, the intrastate border closures facilitated a business-as-normal approach within regions. However, enabling critical food supply to continue to pass through closed borders and keeping wholesale markets operating were considered essential steps to maintain food supplies within Western Australia and particularly the SW region. The restricted travel between cities and within states has hampered food distribution internationally. Workers have lost access to jobs, farmers have had restricted access to inputs, and food imports and exports have reduced or paused [31]. Despite communication challenges, direct messaging and online communication methods have facilitated instant information sharing.

Our study revealed several food business solutions that were borne from the COVID-19 pandemic, such as implementing new ways of working, collaborating to sell produce boxes, restaurants consolidating or changing their menus, schools selling ‘family meals’, and businesses offering customers the chance to bulk buy food items. COVID-safe delivery methods, such as online purchasing and contactless delivery, were considered essential. Collaboration between food supply stakeholders has been considered imperative to improve the visibility of the food supply chain. Research in the Netherlands found increased food supply chain resilience as a result of information sharing and collaborative communication [32]. Importantly, Costa Rican farmers reported that their participation in a farmer “collective” reduced the time demand associated with consumer-direct sales [30]. This approach offers a ‘win-win’ for farmers and consumers, providing greater product diversity for consumers and decreasing the time burden on farmers. Food business adaptability was key in our study, as in the U.S., where smaller producers and distributers adapted their business models for direct sales to the public [12]. Online and contactless payment for food has been reinforced internationally as “the new normal”, with the potential for physical distancing and considering the reduction in business travel [23].

Our findings suggest that ideas, including those for improving infrastructure, such as mobile abattoirs and decentralised packing facilities across regional WA, could represent effective strategies to prepare for future crises. Adaptations to the food supply could be made by connecting smaller retailers to wholesalers via online platforms. Major supermarkets could also offer boxes of ‘essential items’ for vulnerable people to access. Vulnerable community members have been disproportionately impacted by pandemic panic buying, with limited food availability experienced by people who could not afford to stockpile food [29]. Importantly, improving access to locally grown food through co-operatives would act as a protective mechanism. The strengthening of shorter, decentralised food supply chains, such as through increasing local producers, has been recommended internationally [12,31,33]. Kumar et al. have also suggested that moving procurement closer to farmers can reduce pandemic risks [25], while decentralisation can increase food supply chain flexibility [31]. Additionally, blockchain technology has been suggested as a way forward to increase the adaptability and resiliency of food supply chains to future shocks [23].

### Recommendations

Based on our findings, and supported by the literature, recommendations to protect the SWWA food supply during future crises include building social capital by:

- *Cultivating authentic local opportunities for direct food sales*: Government incentives and reduced red tape to support local food distribution and promotion, such as managing e-commerce platforms for farmers and provision of support programs which reduce business adaptation challenges [30], as well as tax relief [34]. Additionally, increase access points for locally grown food [24] and provide educational opportunities for farmers to upskill to sell their products online [30].
- *Boosting collaboration and relationships among food supply stakeholders and consumers:* Cross-promote businesses online through e-commerce platforms [30], increase produce buying power, offer dignified food relief and community ‘goodwill’ for vulnerable people. Establish communication networks among food supply stakeholders to improve knowledge exchange about price increases, availability, access points, and surplus.
- *Supporting local businesses and government to create a food emergency response:* Develop a food security emergency framework that articulates the quantity of food required to be food secure, identifies food access points, and facilitates government and non-government agencies to support food system stabilisation. Broaden the scope and amount of financial support available through the social support system to improve food security [24,25].
- *Planning for future food system disruptions*: Government contingency planning, including provision of farm directories, documenting food needs of institutions from a range of sectors, identifying food supply ‘bottle-necks’ [24], developing a communication strategy to facilitate consumer purchasing changes and mitigate panic buying, and provision of opportunities to access COVID-safe products (i.e., contactless delivery).

Key strengths of this study include being the first in-depth exploration of food supply issues in Australia during the COVID-19 pandemic that captures the perspectives of consumers and multiple food system stakeholders. While we used a convenience sample for Phase 1, it potentially accurately reflects the target population. Limitations include a small sample size (Phase 1) despite using a number of recruitment strategies. We also had a small number of stakeholders from transport and logistics sectors in our Phase 2 sample. The inclusion of more stakeholders from these sectors could have provided a broader range of insights for our study as these were central to food supply chains during the pandemic. Further, the proportion of 51+ year olds (61.3%) in our Phase 1 sample was larger than the 36% within the 50+ years cohort reported at the 2016 Census in Forrest (Commonwealth Electoral Division) [18,35]. Approximately one third (36.4%) of Phase 1 participants were working full time. This was lower than the 2016 Census data for Forrest (South West region) where 52.9% of respondents were employed full time. Approximately 40% of the Phase 1 sample were retired or unemployed, higher than the approximately one-quarter (26.1%) reported by the 2016 Census [18]. A majority of our sample (79.4%) reported a health condition or disability that impacted their ability to undertake physical tasks. This is higher than the 15.3% of Western Australians with a disability [36]. However, South West data was unavailable for a more accurate comparison. These factors collectively limit the generalisability of our findings beyond the SWWA region.

## 5. Conclusions

In conclusion, our study reported substantial changes to the SWWA food supply during the COVID-19 pandemic, with a greater reliance placed on local food by consumers. Businesses adapted by necessity, implementing new ways of working to keep the supply chain moving. Solutions included product substitutions and increased diversification, COVID-safe delivery and collaboration among businesses. Enhancing social capital through such food supply stakeholder collaboration, increasing access to locally produced food, effective contingency planning, developing local infrastructure, improving the media’s role and support for vulnerable community members are actions required to mitigate the impacts of future crises. Future research should ascertain the viability of authentic direct local food sales in regional WA.

## Figures and Tables

**Table 1:** Nodes determined during data analysis.

Node	Child Node
	Local customers (85 coded statements)
Supply chains pre-COVID-19	Corporate customers (75 coded statements)
	Reputation, accreditation (34 coded statements)
	Cooperation, collaboration (5 coded statements)
COVID-19 impact on South West Western Australian food supply chains	Changes in product availability (52 coded statements)
	Changes in distribution modes (46 coded statements)
	Changes in habits and processes (32 coded statements)
Steps taken to maintain the food supply	Keeping supply chain infrastructure operating (37 coded statements)
	Business adaptability (13 coded statements)
	Effective communication (13 coded statements)
Solutions and vision for the future	Educate consumers (42 coded statements)
	Cultivate authentic local markets (24 coded statements)
	Deliver COVID-safe products (22 coded statements)
	Do things differently (19 coded statements)
	Strengthen local relationships (12 coded statements)
	Increase diversification (10 coded statements)
	Boost collaboration (9 coded statements)
	Planning for the future (8 coded statements)
	Remove the middle person (8 coded statements)
	Educate farmers (4 coded statements)

**Table 2:** Phase 1 consumer survey and Phase 2 stakeholder interview participant demographics.

Consumer Survey Respondents		
Variable	Response Categories	Number (Percentage)
Age (Years)	18–30	12 (11.3)
	31–50	29 (27.4)
	51+	65 (61.3)
Gender	Male	18 (17.0)
	Female	88 (83.0)
Adults in Household	1	15 (14.0)
	2	70 (65.4)
	3 or more	22 (20.6)
Children in Household	No children	79 (79.0)
	One or more child	21 (21.0)
Educational attainment	Completed primary or secondary school	14 (13.2)
	Completed technical or further education or university degree	92 (86.8)
Marital status	Married	76 (72.4)
	Single or widowed/separated/divorced	29 (27.6)
Employment status	Full-time work	39 (36.4)
	Part-time work or student and working full- or part-time	25 (23.4)
	Retired, unemployed, employed but not working	43 (40.2)
Disability or health condition	Yes	85 (79.4)
	No	22 (20.6)
Main shopper in household	Yes	91 (85.8)
	No	15 (14.2)
**Food Supply Chain Stakeholder Respondents**		
Sector *	Production	7 (25.9)
	Government	5 (18.5)
	Freight/logistics	2 (7.4)
	Retail	8 (29.6)
	Hospitality	2 (7.4)
	Institution	7 (25.9)
Role *	Primary producer	7 (25.9)
	Retailer, open-air/farmers’ market managers	8 (29.6)
	Hospitality business owner	2 (7.4)
	Local government community development or environmental health staff	5 (18.5)
	Institution coordinator or cook (childcare, aged care, social services)	7 (25.9)
	Logistics or freight managers	2 (7.4)
Duration in role	2 years or less	4 (14.8)
	3–5 years	6 (22.2)
	6–10 years	7 (25.9)
	11–15 years	4 (14.8)
	16–30 years	4 (14.8)
	31+ years	2 (7.5)

* some respondents reported having multiple roles which aligned with multiple sectors.

**Table 3:** Food business and supply solutions during the COVID-19 pandemic reported by consumer and food supply stakeholders.

Solution	Description	Exemplar Quote
*Product substitutions and increased diversification*	Supermarkets buying bulk staples, such as flour, and repackaging into smaller quantities to maintain regional food supply. Supermarkets sold “*less than perfect*” produce. Cafes and fast-food outlets sold staple foods, such as bread, milk and eggs. Food box schemes initiated. Some restaurants consolidated or changed menus to attract more customers for takeaway orders. School canteens sold “*family meals*”. Some businesses offered bulk buying to consumers.	*“More “odd lots” being sold, i.e., vegetables with imperfections”* (Consumer) *“I think that the local cafe type stuff…the person is gonna [sic] pop in and grab a coffee off you. Well, why can’t they grab your, you know, buy some milk, or buy some—a box of vegetables, or a loaf of bread off you at the same time as well.”* (Production sector and hospitality sector, primary producer and hospitality business owner)
*Deliver COVID-safe products*	Food available for purchase online via websites and social media. Offered pre-packed or boxed produce using contactless delivery, pick up, or hygiene practices.	*“They bring all of that produce to me at a warehouse that we’re renting…people start collecting from 9 [a.m.] and we send them a text when it’s ready.”* (Retail sector, Retailer)
*Boost collaboration*	Food businesses shared transportation around the region, or offered assistance through fruit picking. Producers formed buying groups with competing businesses to buy produce at a lower cost and divide it between businesses. A digital network of businesses evolved to supply local food products to local accommodation. Local government promoted food distribution for vulnerable groups on their website and many stakeholders utilised social media.	*“Talk to each other and see if anybody knew of any grower that had X or Y or and we’d make sure we helped each other. We bought big quantities of stuff together and then broke it down and, you know, so that we had better buying power. So, it basically forced us into collaborating with our opposition, if you like.”* (Retail sector, Retailer)

## Data Availability

The datasets generated and analysed during the current study are not publicly available due to ongoing data analysis but are available from the corresponding author on reasonable request.
